# A usability study to improve a clinical decision support system for the prescription of antibiotic drugs

**DOI:** 10.1371/journal.pone.0223073

**Published:** 2019-09-25

**Authors:** H. Akhloufi, S. J. C. Verhaegh, M. W. M. Jaspers, D. C. Melles, H. van der Sijs, A. Verbon

**Affiliations:** 1 Department of Medical Microbiology and Infectious Diseases, Erasmus University Medical Center, Rotterdam, the Netherlands; 2 Department of Internal Medicine, Division of Infectious Diseases, Erasmus University Medical Center, Rotterdam, the Netherlands; 3 Department of Medical Informatics, Center for Human Factors Engineering of Health Information Technology (HIT-Lab), Academic Medical Center, Amsterdam, the Netherlands; 4 Department of Hospital Pharmacy, Erasmus University Medical Center, Rotterdam, the Netherlands; University of Sydney, AUSTRALIA

## Abstract

**Objective:**

A clinical decision support system (CDSS) for empirical antibiotic treatment has the potential to increase appropriate antibiotic use. Before using such a system on a broad scale, it needs to be tailored to the users preferred way of working. We have developed a CDSS for empirical antibiotic treatment in hospitalized adult patients. Here we determined in a usability study if the developed CDSS needed changes.

**Methods:**

Four prespecified patient cases, based on real life clinical scenarios, were evaluated by 8 medical residents in the study. The “think-aloud” method was used, and sessions were recorded and analyzed afterwards. Usability was assessed by 3 evaluators using an augmented classification scheme, which combines the User Action Framework with severity rating of the usability problems and the assessment of the potential impact of these problems on the final task outcomes.

**Results:**

In total 51 usability problems were identified, which could be grouped into 29 different categories. Most (n = 17/29) of the usability problems were cosmetic problems or minor problems. Eighteen (out of 29) of the usability categories could have an ordering error as a result. Classification of the problems showed that some of the problems would get a low priority based on their severity rating, but got a high priority for their impact on the task outcome. This effectively provided information to prioritize system redesign efforts.

**Conclusion:**

Usability studies improve lay-out and functionality of a CDSS for empirical antibiotic treatment, even after development by a multidisciplinary system.

## Introduction

Misuse and overuse of antimicrobial drugs have contributed to the selection of resistant bacteria, which occurs worldwide and has been estimated to contribute to an extra mortality of 10 million people by 2050 [1]. Studies have shown that about 30–50% of antibiotics are being prescribed inappropriately [2–4], and empirically started antibiotics are considered appropriate in only around 60% of the prescriptions [5–7]. Guideline-adherent empirical therapy is associated with a relative risk reduction for mortality of 35% and is therefore described as one of the most important objectives of antimicrobial stewardship programs [8, 9]. The use of a clinical decision support system (CDSS) is a promising method to improve guideline-adherent empirical therapy [10–14]. As part of antimicrobial stewardship, a CDSS can play an important role to prescribe antimicrobial drugs appropriately and according to the guidelines.

CDSSs to support appropriate use of antibiotics have been developed since 1980 [15] and have increased in number in the last years. These systems combine relevant individual patient information with a computerized knowledge base to support decision-making in individual patients. By integrating relevant clinical data and evidence-based guidelines, these systems can help physicians to effectively manage all relevant information necessary for decision making in an increasingly complex clinical practice environment [16]. These systems are considered potentially highly valuable tools to improve clinical decision making and thereby quality of healthcare [15, 16]. CDSSs to support appropriate use of antibiotics target a variety of aspects, such as optimizing antimicrobial dosing [17–19] or supporting antimicrobial de-escalation [20, 21]. Most of these systems however focus on antimicrobial prescribing [15, 22, 23]. It has been shown that CDSS can increase confidence of general practitioners in their antibiotic prescriptions [24]. The systems that are designed to support antimicrobial prescribing in secondary care tend to focus more on a broader population than in primary care, where the systems are often focused on specific syndrome presentation in adults [15]. We have developed a CDSS for empirical antibiotic treatment in hospitalized adult patients, which combines relevant patient information with relevant local antibiotic treatment guidelines. Several other CDSSs for empirical antibiotic prescription have been developed. These CDSS differ on different aspects. Some systems use expert rules to predict the pathogen’s susceptibility to antibiotics, using antibiotic susceptibility profiles from patients with similar characteristics [11, 13, 25], but don’t take into account for example the antibiotic resistance history of the patients of interests or presence of neutropenia [13, 25], like our system does. Others use causal probabilistic networks to predict the probability of a bacterial infection, site of infection and pathogens and their susceptibility to antibiotics. The CDSS we developed generates antibiotic advices based on relevant guidelines. Like many other CDSS for empirical antibiotic therapy input of the physicians was needed in our system for the generation of an antibiotic advice [10–13, 25].

CDSS for empirical antibiotic therapy have shown benefits in terms of improving empirical antibiotic prescribing [10–14]. However, in many of these studies the CDSS was not assessed while or after the end-users, the physicians themselves, used the system[10, 12, 13].

An important issue with the implementation of CDSSs is that they are, until now, not frequently used despite their potential benefits [26]. Studies have shown that poor usability negatively affects CDSS acceptance and effectiveness [27, 28]. Poorly designed CDSS have a negative impact on the use of these systems and can result in medication errors, potentially compromising patient safety [27, 28]. Therefore, the usability of these systems need to be well tested before being implemented in clinical practice. For this purpose we used an augmented classification scheme developed by Khajouei *et al*.[27] to test the usability of our developed CDSS for empirical antimicrobial therapy. This augmented classification scheme combines the User Action Framework (UAF), a standardized validated classification framework, with severity rating of the usability problems and the assessment of the potential (clinical) impact of these problems on the final task outcomes [27]. To our knowledge no other studies have assessed and described the usability of a developed CDSS for antimicrobial drug prescription using this systematic framework.

The aim of this study was to detect usability problems in our developed CDSS for empirical antimicrobial therapy, to rate the severity of these problems, and to determine the impact on the task outcome.

## Materials and methods

### Setting

This study was conducted at the Erasmus MC, University Medical Center in Rotterdam, the Netherlands, a tertiary care center with all medical specialties available. The Erasmus MC uses an electronic health record (EHR) with integrated computerized prescriber order entry (CPOE) which was introduced in December 2001.

### Clinical decision support system (CDSS)

**Fig 1:**
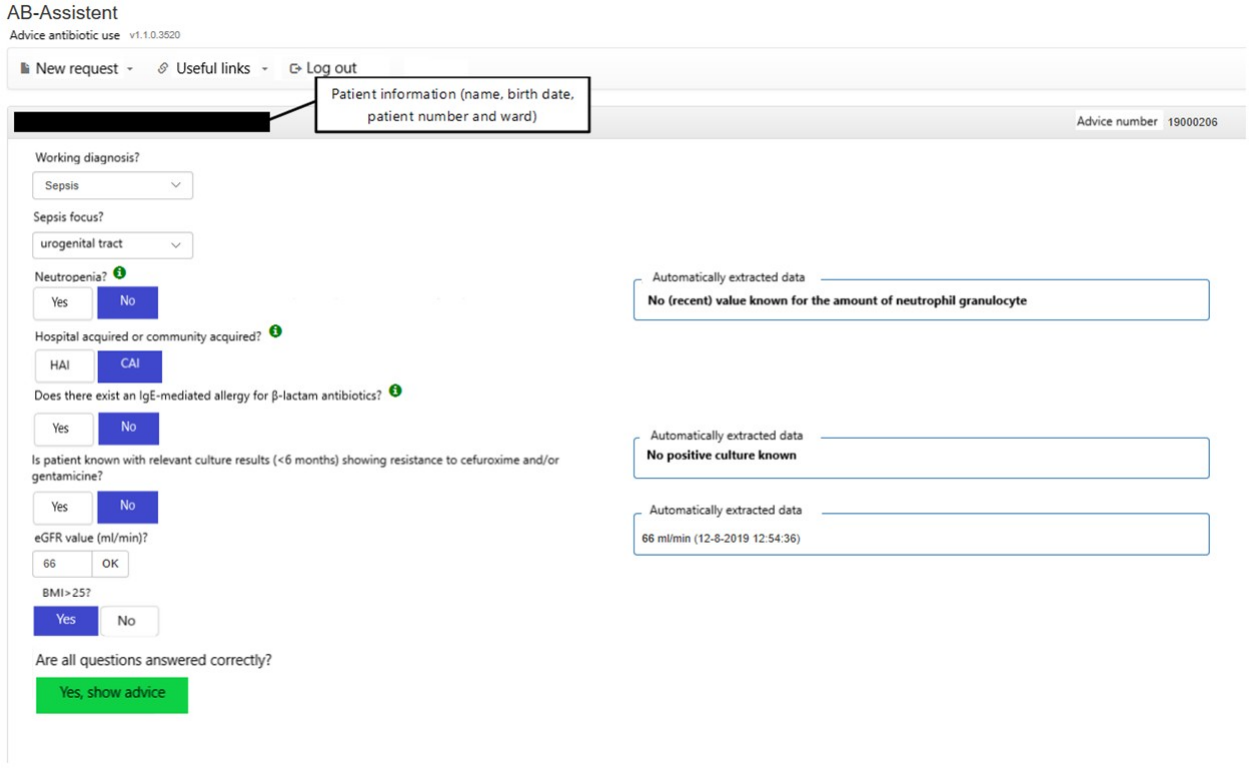
The developed CDSS, which combines relevant electronic patient information with relevant local antibiotic treatment guidelines.

A rule-based CDSS for empirical antibiotic treatment in adult patients was built as a web application by a multidisciplinary team of clinical experts and information and communications technology (ICT) professionals (Fig 1). The system has been developed to give empirical antibiotic treatment advice for the following infections: pneumonia, sepsis, urinary tract infections, meningitis and secondary peritonitis.

The developed CDSS combines relevant electronic patient information derived from the Erasmus MC electronic medical record (such as kidney function, microbiological results from the previous 6 months and presence or absence of neutropenia) with relevant local antibiotic treatment guidelines, which are in line with national guidelines (http://www.swabid.nl). The result is an indication driven advice that is patient specific and in accordance with current guidelines. Relevant patient information were as much as possible automatically extracted from our HER, to which the CDSS was connected. To generate an appropriate antibiotic advice some information input, which could not be automatically extracted from our hospital information system, had to be entered manually by the user (for example the working diagnosis).

### Testing the usability of the CDSS

To identify usability problems in the design of a CDSS, different usability evaluation methods can be used. One of the methods to assess usability is the use of surveys, for example the often used System Usability Scale [29, 30]. This is a validated survey instrument, which consists of 10 items that have to be rated on a 5-point agreement scale [29]. It is a relative quick and easy instrument to use and it covers areas such as user satisfaction, efficiency of use and system effectiveness [31]. This method has already been used for assessing the usability of a CDSS for antibiotic prescription [24, 32]. We did not use this survey instrument, because it does not provide insight in details or causes of identified problems. Other usability evaluation methods, which are often used are the heuristic evaluation, the cognitive walkthrough and the think aloud method [33]. The first two mentioned methods are expert-based methods, whereas the think aloud method is a user-based method. With the heuristic evaluation potential usability problems are uncovered using heuristics, which are recognized usability principles [34]. An example of a heuristic is ‘provide help and documentation’. We did not use this method because the used heuristics are often very generally described, making them multi interpretable, resulting in different outcomes. This method is also highly dependent on skills and experience of the evaluator to improve the results overall [33]. With the cognitive walkthrough a usability expert simulates a new user by walking through the system step-by-step using typical tasks and details about the user’s background. This is a really structured approach, however it is a very tedious method, time consuming and the results are affected by the task description and given details about the user’s background [33]. We have chosen to use the think aloud method [35], because this method is a very rich source of data regarding usability problems. This is a user-based usability evaluation method where participants have to verbalize their thoughts during the execution of a set of specified tasks. It provides detailed insight into usability problems actually experienced by end-users of the system. Of added value is that this method provides insight in the causes of the identified problems. The verbal data are used to evaluate the system’s design on usability flaws.

The usability study was performed in 2 steps. During the first step residents completed tasks using the CDSS and during the second step the usability of the system was assessed using the data that were collected during the first step. During the first step 15 medical and surgical residents were invited by e-mail to participate in the study. Residents were invited as participants in this study, because they are the intended users of the CDSS. Selection of residents was based on: I) diversity in discipline, II) prescribers of different antimicrobial drugs, III) years of residency and IV) not being involved in the development or analysis of the CDSS. Eight residents (3 from internal medicine, 2 from surgery, 2 from medical microbiology, 1 from neurology) participated in this study. The residents were on average 31 years old, and in their first to 6th year(s) of residency and 4 were female.

The residents were given a short demonstration of the CDSS before the usability test. The CDSS was not used in hospital before the study. Four test cases were developed based on real life clinical scenarios (for description of these test cases see S1 Table). The test cases were assessed on correctness, completeness and clearness by clinical experts in our study team. During the usability test, participants were asked to complete the tasks of antimicrobial drug prescription while an observer watched, listened with minimum interruption and recorded (audiotaped and videotaped) the entire test session. All participants used the same web browser during the test and completed all four test cases.

### Evaluating the usability of the CDSS

During the second step usability of the system was assessed using the data that were collected during the first step. This assessment was done by 3 unblinded evaluators, a physician, a hospital pharmacist experienced in clinical decision support and a researcher in the field of quality. Assessment was done by 2 evaluators, independently of each other. One of these primary evaluators had not been involved in the development of the CDSS. Disagreements in the sets of usability problems were resolved in discussion with a third evaluator. For this assessment an augmented classification scheme developed by Khajouei *et al*. was used [27]. This augmented classification scheme combines the User Action Framework (UAF), a standardized validated classification framework, with severity rating of the usability problems and the assessment of the potential (clinical) impact of these problems on the final task outcomes [27]. Each cycle of the user system interaction, which contains 4 phases (planning, translation, physical actions and assessment) was assessed. Planning is the phase of the user system interaction cycle including all cognitive actions by users to determine *what* to do. In the translation phase users determine *how* to accomplish the intentions that emerge during the planning phase. The phase in which the actions are being carried out by manipulating user interface objects is the physical action phase. The assessment phase is about the perception, interpretation and evaluation of the resulting system state by the user. Usability problems were identified using the videotapes of the cases and classified under different subcategories to the most detailed level using the UAF hierarchy [27]. Severity rating of usability problems was performed using the Nielsen’s classification [35]. This severity rating is based on the (potential) impact of the problem on the users, the (potential) persistence of the problem and the frequency with which a problem (might) occur(red).

## Results

**Fig 2:**
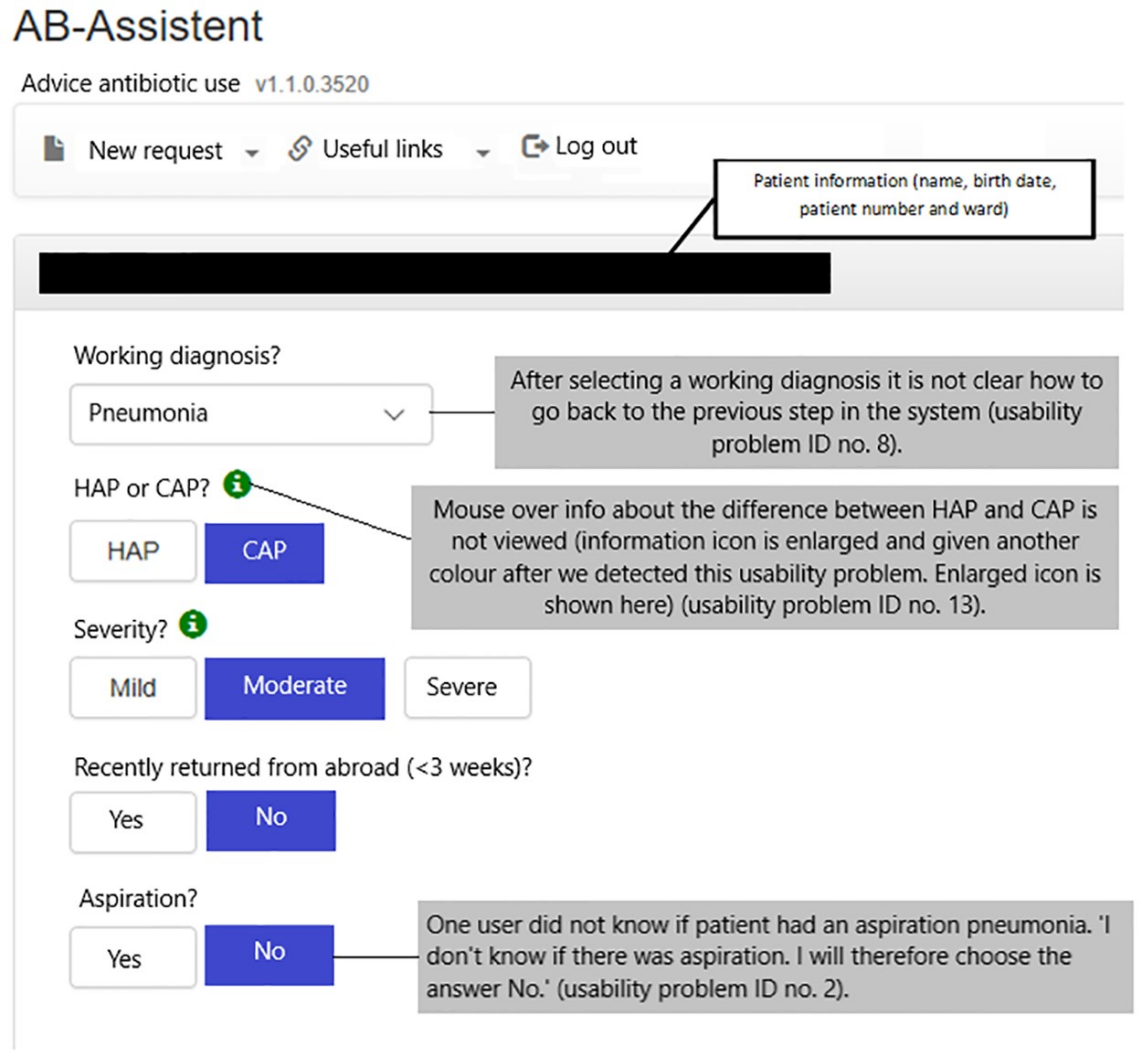
Some usability problems in the CDSS for empirical antibiotic therapy.

**Fig 3:**
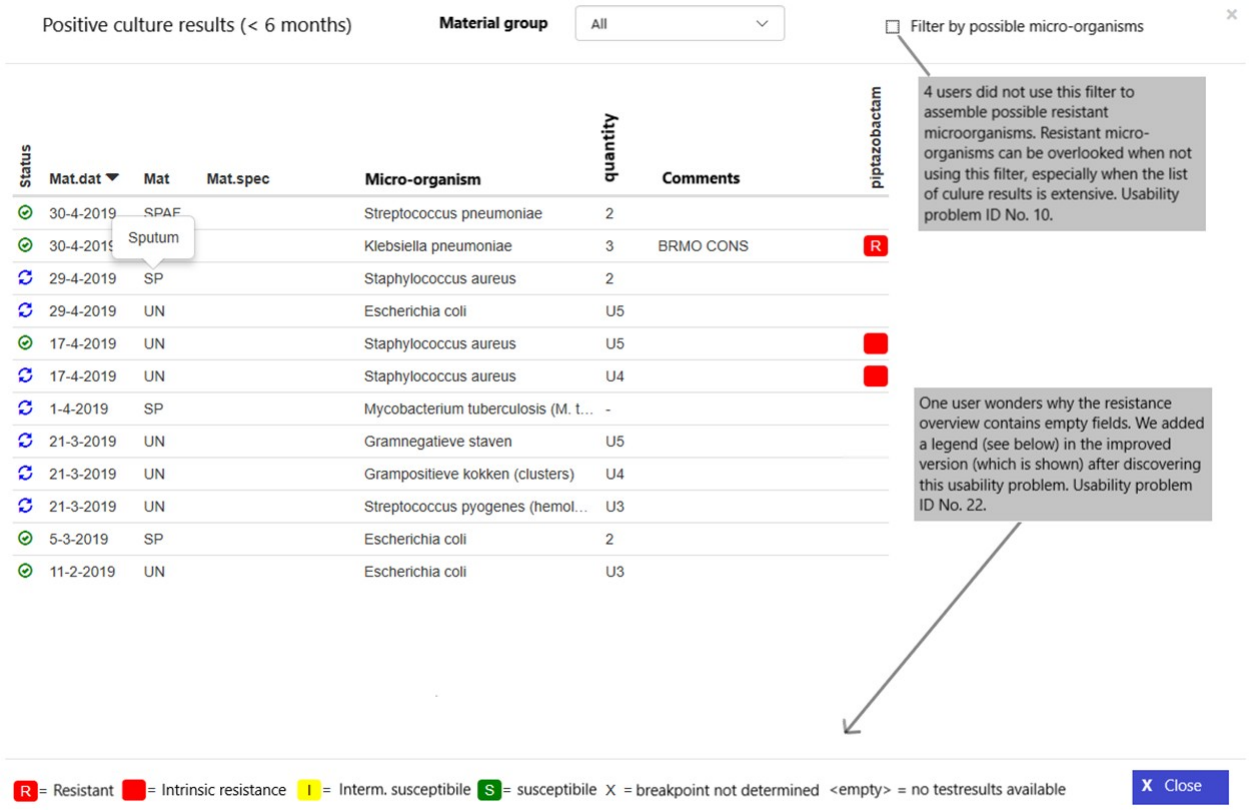
The resistance viewer in the CDSS for empirical antibiotic therapy and illustration of 2 usability problems.

**Fig 4:**
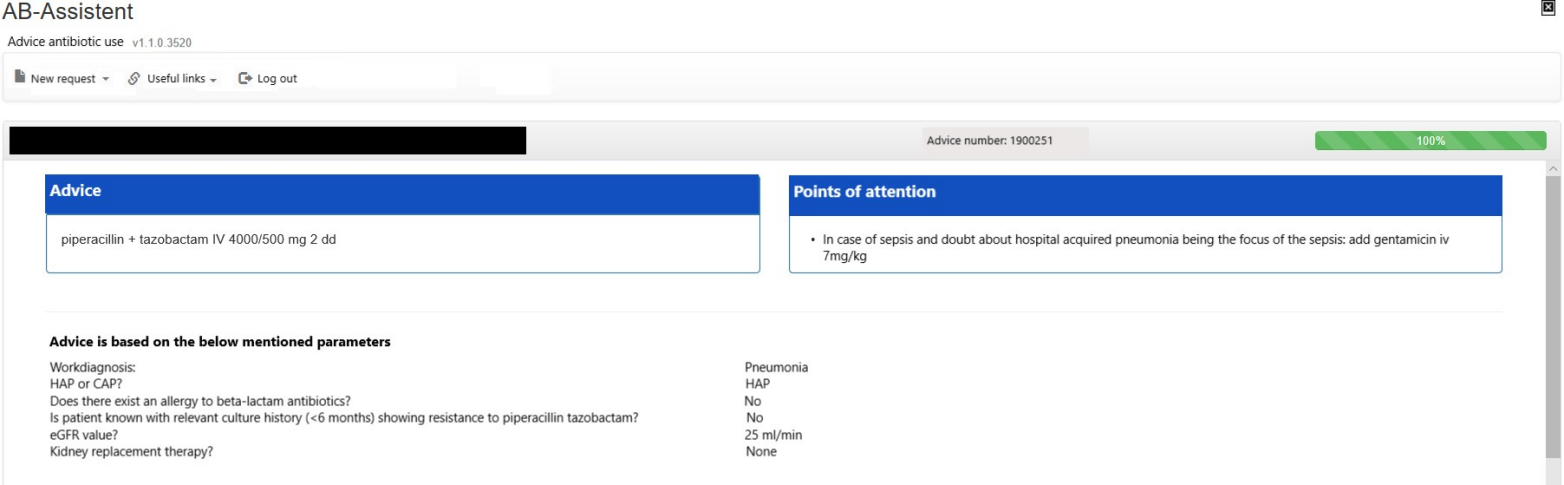
Final screen of the CDSS for empirical antibiotic therapy with a patient specific antibiotic advice.

In total, 51 usability problems were identified in the usability evaluation studies, of which 7 in the planning phase (Table 1), 28 in the translation phase (Table 2), 4 in the physical actions phase (Table 3) and 12 in the assessment phase (Table 4). These 51 usability problems could be grouped into 29 different categories. A description and illustration of some of these usability problems can also be found in Figs 2 and 3. Fig 4 shows the final screen with a patient specific antibiotic advice generated.

**Table 1:** Usability problems in the UAF planning phase with their severity and potential effect on task outcome.

ID No.	Level 1	Level 2	Level 3	Level 4	Description of usability problem	No.^1^	Severity	Identifying potential outcomes^2^
1	Planning	Users model of the system	Users ability to determine what to do first		It is not immediately clear for the user which diagnosis has to be chosen in case of urosepsis (two possible pathways). The first information input that had to be entered manually by the user is the diagnosis. This is done by selecting one of the diagnosis in a drop down menu. For the diagnosis urosepsis the user has two possible pathways, namely the user can select sepsis, with sepsis focus urogenital tract or high urinary tract infection.	3	2	None (both pathways same result)
2		Goal decomposition	Users ability to determine what to do next		User has to answer if patient has an aspiration pneumonia. Because user doesn’t know user chooses the answer ‘no’. The option ‘unknown’ does not exist in the system.	1	0	Wrong antibiotic
3		Goal decomposition	Users ability to determine what to do next		Information is missing about what has to be filled in when the existence of neutropenia is unknown.	2	2	Wrong antibiotic
4		Users knowledge of system state, modalities.			When entering a new patient identification number nothing happens.	1	2	None

^1^The number of usability problems with the same classification path, in the interaction of different or the same user with the system.

^2^The mentioned outcomes are potential and did not have to occur.

**Table 2:** Usability problems in the UAF translation phase with their severity and potential effect on task outcome.

ID No.	Level 1	Level 2	Level 3	Level 4	Description of usability problem	No.^1^	Severity	Identifying potential outcomes^2^
5	Translation	Existence	Existence of a way		The user calculates, or even guesses, the BMI with a calculator outside the system.	8	3	Wrong dosage
6		Existence	Existence of a way		The user calculates the needed dosage of gentamicin with a calculator outside the system. The user mentions that it would be helpful if the dosage is calculated by the system.	3	4	Wrong dosage
7		Existence	Existence of a way		User has to fill in the weight and body height of the patient. This information is not automatically retrieved from the hospital information system. User expresses the wish that dosage of antibiotic is calculated with automatic retrieved weight and body height.	1	2	Wrong dosage
8		Existence	Existence of a way		The user has to select a working diagnosis from a drop down menu. After selecting a working diagnosis it is not clear how to go back to the previous step in the system.	1	3	None
9		Presentation	Perceptual issues	Noticeability	The user does not know (immediately) how to perform a request for another patient.	4	1	None
10		Presentation	Perceptual issues	Noticeability	The user does not use the filter to assemble possible resistant micro-organisms in the resistance overview profile, which shows the culture history (if filter is not being used resistant micro-organisms can be overlooked).	1	0	Wrong antibiotic
11		Presentation	Perceptual issues	Noticeability	User does not view the overview of AST^3^, and the materials (for example sputum, urine and blood) in the resistance overview profile which shows the culture history.	2	0	Wrong antibiotic
12		Presentation	Perceptual issues	Noticeability	Overview of resistance, which shows the culture history, is not seen immediately.	1	2	Wrong antibiotic
13		Presentation	Perceptual issues	Noticeability	Mouse over info about difference between HAP^4^ and CAP^4^ is not viewed.	3	2	Wrong antibiotic
14		Presentation	Perceptual issues	Noticeability	Mouse over info about severity of pneumonia is not viewed. Argument for severity classification is not correct.	3	2	Wrong antibiotic
15		Presentation	Perceptual issues	Noticeability	User overlooks the information provided about the ESBL^5^ positivity of the patient.	1	0	Wrong antibiotic

^1^ The number of usability problems with the same classification path, in the interaction of different or the same user with the system.

^2^The mentioned outcomes are potential and did not have to occur.

^3^ AST: Antibiotic Susceptibility Tests.

^4^HAP: hospital acquired pneumonia, CAP: community acquired pneumonia.

^5^ ESBL: extended spectrum betalactamase

**Table 3:** Usability problems in the UAF physical action phase with their severity and potential effect on task outcome.

ID No.	Level 1	Level 2	Level 3	Level 4	Description of usability problem	No.^1^	Severity	Identifying potential outcomes^2^
16	Physical actions	Manipulating objects	Physical layout		To view the complete resistance overview, which shows the culture history, the user has to scroll down in the resistance viewer. The user does not scroll down in this viewer.	1	2	None
17		Manipulating objects	Preferences and efficiency		User wants to review the culture history, when advice is generated, but this is not possible (functionality not available). User thinks this is not convenient, because the user wishes to review this history while consulting an infectious diseases consultant.	1	4^3^	None
18		Manipulating objects	Preferences and efficiency		Physician mentions that she misses a button (button does not exist in the system). There is only the possibility to answer ‘yes’ or ‘no’ on the question if patient has been abroad. She mentions there has to be a button ‘unknown’.	1	2	None
19		Perceiving physical objects	Perceiving objects as they are being manipulated		The user tries to click through the resistance viewer, which shows the culture history. This is not possible (this functionality is not available in the system)	1	1^3^	None

^1^The number of usability problems with the same classification path, in the interaction of different or the same user with the system.

^2^The mentioned outcomes are potential and did not have to occur.

^3^.The difference in severity between these 2 usability problems stands out. The usability problem ‘The user tries to click through the resistance viewer, which is not possible’ is scored as 1 (cosmetic problem), because it has a low impact on the user interaction, the problem only occurred once and is an usability problem which is not persistent.

**Table 4:** Usability problems in the UAF assessment phase with their severity and potential effect on task outcome.

ID No.	Level 1	Level 2	Level 3	Level 4	Description of usability problem	No.^1^	Severity	Identifying potential outcomes^2^
20	Assessment	Feedback	Content and meaning	Completeness and sufficiency of meaning	The user questions what to do with ‘Advice number’.	1	0	None
21		Information display	Content and meaning	Error avoidance	The message ‘No relevant cultures known’ is confusing. This message only refers to cultures in this hospital	2	1	Wrong antibiotic
22		Information display	Content and meaning	Error avoidance	The user wonders why the resistance overview includes empty fields.	1	3	Wrong antibiotic
23		Information display	Content and meaning	Error avoidance	The advice does not clearly indicate for what antibiotic the trough level has to be determined.	2	2	Determining medication dosage for the wrong antibiotic
24		Information display	Content and meaning	Error avoidance	Physician reads essential information accompanying the advice, but prescribes the wrong antibiotic which is contrary to this information.	1	2	Wrong antibiotic
25		Information display	Content and meaning	Layout and grouping	The final advice already appears earlier under a mouse over (which can be confusing).	1	2	None
26		Information display	Content and meaning	Layout and grouping	The resistance overview displays the results of a bone marrow biopsy, which confuses the physician.	1	2	Wrong antibiotic
27		Information display	Existence	Human memory aids	It is not clear whether the resistance viewer also takes resistance into account determined in other hospitals.	1	0	None
28		Information display	Presentation	Perceptual issues > noticeability	Not clear whether the user realizes the Gentamicin doses has to be adjusted.	1	2	Wrong dosage
29		Information display	Presentation	Perceptual issues > noticeability	The physician does not read the text which states that the Gentamicin dose has to be adjusted in case of a too high body mass index.	1	3	Wrong dosage

^1^The number of usability problems with the same classification path, in the interaction of different or the same user with the system.

^2^The mentioned outcomes are potential and did not have to occur.

### Planning

Seven (14%) of the identified usability problems were found in the planning phase of user-system interaction (Table 1). The usability problems in this phase were mainly caused by the user’s difficulties in choosing the correct diagnosis (two possible pathways), lack of a third option such as an ‘unknown’ button, and perceived lack of information (user is not provided with information about the system state, when entering a new patient identification number fails).

Classification of the problems with the augmented scheme showed that some of the problems would get a low priority based on their severity rating, but got a high priority for their impact on the task outcome. For example, the severity of the usability problems leading to the prescription of wrong antibiotics was rated as minor or no problem while the impact of prescribing the wrong antibiotic can be high.

### Translation

Twenty-eight (55%) of the usability problems concerned the translation phase (Table 2). The usability problems in this phase were mainly caused by the fact that the mouse over functions were not noticed or correctly used, and that extra patient information (culture results) were not noticed by users. Also, the needed doses of gentamicin and the BMI were calculated with a calculator outside the system or guessed, leading to wrong dose advices.

Most usability problems had low severity ratings. Only one usability catastrophe (severity rating of 4) was observed when the gentamicin dose had to be calculated and users did look for a calculator, which was not available in the CDSS. The users expressed the need for a calculator. Not only the usability problem had a high severity rating of 4, but the impact of the problem is high too.

### Physical actions

Four (8%) of the usability problems were encountered in the phase of physical actions (Table 3). One of these usability problems was caused by the layout of an object, for instance the scroll down button that had to be used. Another usability problem in this phase was the lack of user control over screen objects as these objects were being manipulated. For example, the user tried to click through the resistance viewer, but this was not possible. Two usability problems in this phase concerned the failure of the system to meet specific preferences of users for performing physical actions. One of these problems was the inability to review the culture history when the CDSS had generated an advice. This problem was rated as severity 4, although it would not lead to a wrong medication selection. The user indicated that this problem had a great impact on him, because he wanted to review the culture history during the consultation of an infectious disease specialist when an advice is generated.

### Assessment

In total 12 (24%) of the 51 identified usability problems were classified in the assessment phase (Table 4). These problems concerned the existence, presentation, content and meaning of system feedback about the course of the user-interaction and the display of information resulting from users’ actions.

Not all the problems, that influence the outcome were highly severe problems since three of the problems potentially resulting in wrong antibiotic selection were assigned severity 2, and one problem assigned severity 1. The UAF classification showed that 4 (33%) of the problems concerning the assessment phase of interaction were caused by absent or unclear information displayed after the user’s action to avoid errors. The remaining eight (67%) problems in this phase were caused by the absence, poor presentation or noticeability of information or feedback displayed after the users’ actions.

A general striking finding was that four users indicated that they would not indiscriminately follow the advice given, because they were aware of the fact that the CDSS was recently developed and might contain errors.

## Discussion

With the augmented scheme for classifying and prioritizing usability problems described by Khajouej *et al*. (2011) we found 51 usability problems in different phases of the user system interaction. Most usability problems were found in the translation phase (55%). Testing the usability of a CDSS with this scheme proved to be a simple, but effective way to identify usability problems and prioritize system redesign efforts. With the use of the augmented UAF the existence of usability problems, that were not foreseen, were identified. Also, the frequency of problems of CDSS use, the severity and potential impact of these problems on task outcome were identified. Assessing usability of a CDSS is important to increase the chance of its adoption.

This study is the first to report usability testing of a CDSS for empirical antibiotic treatment in adult patients using the systematic framework developed by Khajouei *et al*.[27]. A strength of this study is that we used the standardized and validated UAF, augmented with a severity rating based on Nielsen’s classification and the assessment of potential effect of the problem on the task outcome. This approach enables the report of existing usability problems in an accurate, complete and consistent way. This is needed for guiding and prioritizing system redesign efforts. Some limitations of this study should also be recognized. Firstly, we could have missed usability problems because of the small group of participants. However, the group of 8 participants was a well representative group, composed out of residents from different disciplines and different years of residence. In addition, about 80% of usability problems can be discovered with only 8 participants and the more severe a problem is, the more likely it will be uncovered within the first few subjects [36–38]. Studies to determine the optimal number of participants for a usability study have shown that the complexity of the study itself is an important factor to consider [37, 38]. Because the tasks the user had to perform in our study were simple and really straightforward we think that 8 participants were enough to detect most usability problems. Another limitation is that participants may have modified their behavior and reported thoughts in response to their awareness of being observed during the usability test. This so-called Hawthorne effect is inherent to simulated usability studies and not possible to rule out [39]. Because all participants were residents, lack of experience could have contributed to the existence of certain usability problems. These problems will probably not be experienced by medical specialists. However, given the fact that residents and specialists with not much experience in antibiotic prescribing, will be the mainly end-users/are the intended users of the CDSS, these problems are important to discover and take into account in the system redesign.

In this usability study participants completed tasks of antimicrobial drug prescription using four prespecified test cases which were based on real life clinical scenarios. In a setting with real patients, the physician know his or her patients and can answer certain questions about a patient better than with the use of a prespecified case, such as the question if the patient has neutropenia. It could therefore be that certain usability problems will not exist or exist less in a setting with real patients which are known by the user. However, this only applies for usability problems where continuing in the system is not possible without knowing certain information (for example neutropenia or if the patient has been abroad). In addition other usability problems could also be revealed when using this CDSS in real clinical conditions.

With this study we found that some of the residents did not follow the advice that was given by the CDSS without thought. They were aware of the fact that the CDSS was recently developed and might contain errors. We also found that time has been invested in the development of functionalities, which were not (optimally) used. An example is presenting mouse over information in addition to certain questions, providing relevant information to the user. Our study showed that these help texts were often not used, which prompted us to enlarge the information icon that makes this help text appear when moving the cursor towards the information icon. Also, simple improvements such as the introduction of a calculator and patient information that is automatically retrieved from the hospital information system such as weight and body height are worthwhile investments. Another simple modification we made to the CDSS is the introduction of a new option, namely the option to review the culture history in the final screen when an antibiotic advice is generated. With these alterations in the system design we made the CDSS more specific to users’needs. For ultimate system usability, iterative usability evaluation during the development and implementation of CDSS are important [28, 40, 41].

## Conclusion and recommendations

Our study revealed several usability problems in different phases of the interaction between the intended user and a CDSS developed for empirical antibiotic treatment, the severity of these problems and the impact on the task outcome. It shows that even though the CDSS has been developed by a multidisciplinary team of clinical experts and ICT professionals, many usability problems can exist that are not foreseen. Assessing usability before CDSS implementation is recommended for improving CDSS adoption, effectiveness and safety. When designing a CDSS the following elements have to be considered to avoid usability problems:

- ‘When a question has to be answered with a yes or no also provide the answer ‘unknown’. If answering with yes or no is necessary for the system to generate an advice, provide users with this information.
- Make it easy to do right by providing calculators for everything that has to be calculated (the recommended dosage of an antibiotic drug, BMI etc.).
- Retrieve as much information as possible automatically from the hospital information system.
- Pay attention to the noticeability of relevant information (for example mouse over info with relevant explanatory information/definitions, resistance overview with information that is relevant for the final antibiotic advice).
- Provide users with information that is clear and as specific as possible and avoid reporting of irrelevant, confusing information.

## Supporting information

S1 Table The four test cases that were used to test the usability of the CDSS. (DOCX)
