# Supplementary material for: A usability study to improve a clinical decision support system for the prescription of antibiotic drugs
Source: PLoS One. 2019 Sep 25;14(9):e0223073. doi: 10.1371/journal.pone.0223073 (PMC6760771; doi:10.1371/journal.pone.0223073)
Supplement: S1 Table — (DOCX) [file pone.0223073.s001.docx]

| Test case 1 | Man, 45 years old (height 1,80m, weight: 100kg), presents to your emergency room with symptoms of an urosepsis. Patient has an impaired kidney function (eGFR 66ml/min). You decide to admit patient and start antibiotic therapy. |
| --- | --- |
| Test case 2 | Female, 70 years old (height 1,55m, weight: 65kg), is transferred from nursing home *Leeuwenhoek* to your hospital because of a pneumonia. She has an impaired kidney function (eGFR=25ml/min), but does not use any renal replacement therapy. |
| Test case 3 | Female, 64 years old (height 1,60m, weight: 80kg) is admitted to your department with a suspected urinary tract infection. She uses the medicine Tacrolimus, because of a kidney transplantation she underwent 5 years ago. She has a 40 degree fever. You would like to prescribe antibiotic therapy. Patient does not have any allergies or a history of antibiotic resistance. She has a good kidney function. |
| Test case 4 | Man, 48 years old (height 1,65m, weight: 80 kg), is admitted to your hospital with a intracerebral hematoma, complicated by oedema, for which an external ventricular drain is placed.  Patient is transferred to your department and develops meningitis with cerebrospinal fluid leakage. Patient does not have any allergies or a history of antibiotic resistance. Patient has a good kidney function. You would like to prescribe antibiotic therapy. |
